# EMOTIONAL FUNCTIONING IN PEOPLE WITH DEMENTIA AND CAREGIVER AFFECT DURING DYADIC INTERACTIONS

**DOI:** 10.1093/geroni/igac059.2813

**Published:** 2022-12-20

**Authors:** Hannah Cozad, Yuxuan Chen, Casey Brown, Julian A Scheffer, Kuan-Hua Chen, Robert W Levenson

**Affiliations:** Georgetown University, Oakland, California, United States; Stanford University, Stanford, California, United States; University of California, Berkeley, Berkeley, California, United States; University of California, Berkeley, Berkeley, California, United States; University of California, Berkeley, Berkeley, California, United States; University of California, Berkeley, Berkeley, California, United States

## Abstract

Dementia caregivers can experience negative affect when interacting with their care recipients. However, few studies have examined the specific factors that predict caregiver negative affect in this dyadic context. We hypothesized that deficits in care recipients’ emotional functioning would be associated with increased intensity of caregivers’ negative affect during interactions with their care recipient. Caregivers (Nf100) reported on two aspects of their care recipients’ emotional functioning: (1) emotion recognition (the ability to recognize other people’s emotions), and (2) negative emotional reactivity (the ability to generate negative emotional responses). Dyads then visited the laboratory and engaged in a 10-minute conversation about an area of conflict in their relationship. Caregivers then watched recordings of their conversation while rating the valence and intensity of their experienced affect using a rating dial. We used these ratings to quantify changes in caregivers’ emotional valence across the course of the conversation. Caregivers of care recipients with greater deficits in emotion recognition demonstrated greater increases in negative affect across the conversation. In contrast, care recipients’ negative emotional reactivity was not related to changes in the valence of caregivers’ affect across the conversation. Findings remained significant even after accounting for caregiver baseline valence ratings, biological sex, and age, as well as the care recipients’ diagnosis and level of cognitive impairment. Results reveal the important role that care recipients’ deficits in emotion recognition play in caregivers’ emotional lives. Caregivers’ negative affect may be more likely to increase when their care recipient has deficits in emotion recognition.

